# Ultrafast Photon-Induced Tunneling Microscopy

**DOI:** 10.1021/acsnano.1c06716

**Published:** 2021-11-01

**Authors:** Manish Garg, Alberto Martin-Jimenez, Yang Luo, Klaus Kern

**Affiliations:** † Max Planck Institute for Solid State Research, Heisenbergstrasse 1, 70569 Stuttgart, Germany; ‡ Institut de Physique, Ecole Polytechnique Fédérale de Lausanne, 1015 Lausanne, Switzerland

**Keywords:** ultrashort pulses, atomic space−time resolution, angstrom−femtosecond resolution, 4D tunneling microscopy, ultrafast optical STM techniques

## Abstract

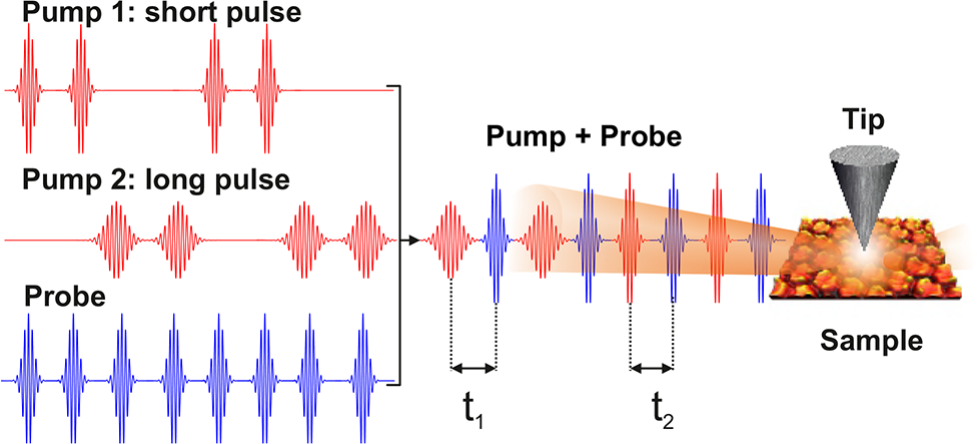

Unification of the techniques of ultrafast science and scanning tunneling microscopy (STM) has the potential of tracking electronic motion in molecules simultaneously in real space and real time. Laser pulses can couple to an STM junction either in the weak-field or in the strong-field interaction regime. The strong-field regime entails significant modification (dressing) of the tunneling barrier of the STM junction, whereas the weak-field or the photon-driven regime entails perturbative interaction. Here, we describe how photons carried in an ultrashort pulse interact with an STM junction, defining the basic fundamental framework of ultrafast photon-induced tunneling microscopy. Selective dipole coupling of electronic states by photons is shown to be controllable by adjusting the DC bias at the STM junction. An ultrafast tunneling microscopy involving photons is established. Consolidation of the technique calls for innovative approaches to detect photon-induced tunneling currents at the STM junction. We introduce and characterize here three techniques involving dispersion, polarization, and frequency modulation of the laser pulses to lock-in detect the laser-induced tunneling current. We show that photon-induced tunneling currents can simultaneously achieve angstrom scale spatial resolution and sub-femtosecond temporal resolution. Ultrafast photon-induced tunneling microscopy will be able to directly probe electron dynamics in complex molecular systems, without the need of reconstruction techniques.

The quest to achieve atomic resolution both in real space and real time simultaneously has led to numerous efforts of integration of ultrashort laser pulses with a scanning tunneling microscope (STM) over the past 30 years.^1−16^ Integration of high-energy, low-repetition-rate optical pulses at the STM junction leads to thermal instabilities, i.e., periodic contraction and expansion of the nanotip.^17,18^ This undesirable artifact leads to a dramatic reduction of the spatial resolving capability of the STM, besides generating an artificial laser-induced tunneling current. Low-energy (few pJ to few nJ), high-repetition-rate (∼80 MHz) optical near-infrared (NIR) laser pulses with a duration lasting only a few femtoseconds have been successfully integrated with an STM, preserving its angstrom resolving capability.^7^

Owing to the high photon energy (∼1.5 eV) and high photon flux (>10^6^ photons per pulse) of low-energy (∼50 pJ) optical NIR pulses, electrons in an STM junction can absorb one, two, or three photons to tunnel to the other side of the junction.^8,19^ Alternatively, at high field strengths, the tunneling barrier of the STM junction can be modified by the laser pulses, enabling electrons to tunnel to the other side of the junction.^4,6,8^ These two different kinds of electron tunneling processes are referred to as photon-driven and field-driven, respectively. With optical NIR laser pulses one can access both kinds of tunneling regimes. On the contrary, while using low-frequency laser pulses such as a few hundred fs long THz pulses, only field-driven tunneling is possible,^4,6^ whereas, in the photon-driven regime, the interaction of laser pulses is only in the weak field, where the laser pulse acts only as a small perturbation. Selective excitation of molecular states and ensuing dynamics can be controlled in photon-driven STM junctions.^7^

Experiments performed using the pioneering technique of shaken-pulse-pair excitation^1,20,21^ (SPPX) have shown that the time scale of thermal effects, i.e., expansion or contraction of the nanotip of the STM on integration of low-energy optical NIR pulses (∼25 pJ), is ∼100 ns or longer.^20^ Thus, a laser pulse train where individual pulses are separated by ∼11 ns (repetition rate ∼ 80 MHz) will generate a constant heat load at the STM junction and will not lead to any thermal artifacts. Moreover, the relevant time scale of the dynamics probed with ultrashort laser pulses (τ ∼ 6 fs) is from few fs to few ps.^22−24^ This time scale covers the time window where ultrafast dynamics in a molecule unfolds, such as electronic transitions and vibrational motion of nuclei.

The integration of laser pulses is supposed not to strongly perturb the standard operation of the STM; hence, the laser-induced tunneling current must be a very small fraction of the DC tunneling current. This poses the challenge of how to lock-in detect the very small tunneling currents generated by the laser pulses at the STM junction.

The most straightforward approach is to perform amplitude modulation of the laser pulses, which is to periodically turn on and off the intensity of the laser pulses at the STM junction and lock-in detect the laser-induced tunneling current at the modulation frequency.^25^ The technique of amplitude modulation of laser pulses at the STM junction has recently unraveled the energy flow between quantum dots and florescence at the nanoscale.^26,27^ Nevertheless, this approach may lead to nonlinear heating of the STM junction which is sensitive to the modulation frequency of the laser pulses,^8^ generating an artificial laser-induced tunneling current. In order to overcome this fundamental obstruction on integrating laser pulses at the STM junction, here we introduce three innovative techniques to lock-in detect the laser-induced tunneling current without the need of amplitude modulation. Dispersion, polarization, and frequency modulated tunneling microscopies are introduced in the following sections in respective order. Thermal effects at the STM junction arising due to amplitude modulation are discussed in the last section. All of these modulation techniques are realized in the photon-driven tunneling regime, which dominates the interaction of laser pulses at the STM junction at low intensities (<10^12^ W/cm^2^), where the Keldysh parameter is greater than one.^8,24^ The bandwidth of the ultrashort laser pulses used in the current work spans from 650 to 1050 nm, the pulse energies varied from a few pJ to a few nJ, and the repetition rate of the laser pulses is ∼80 MHz. A schematic illustration of the integration of ultrashort laser pulses at the STM junction is shown in Figure 1a.

**Figure 1:**
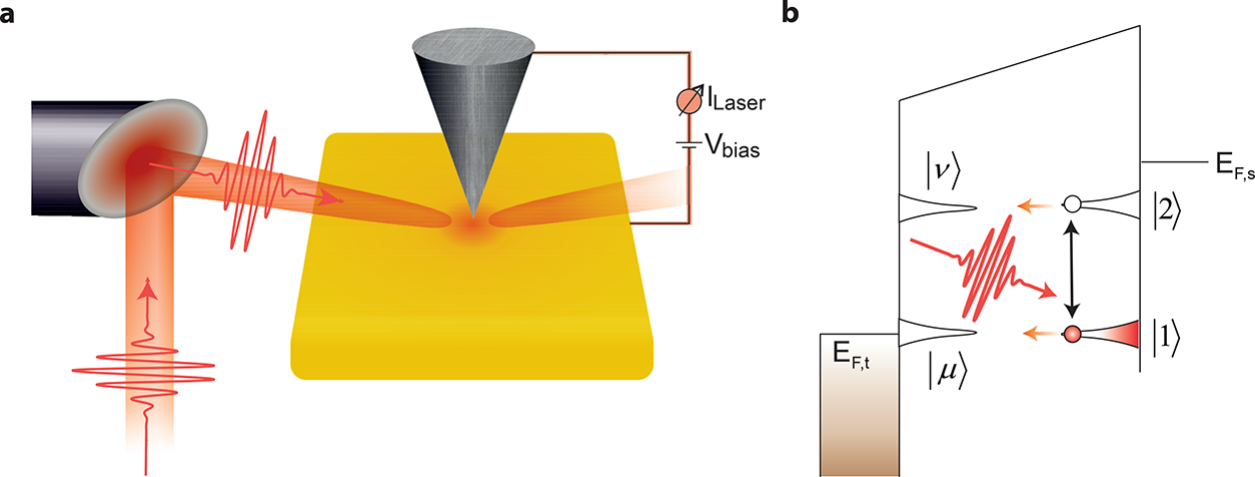
Schematic of the experimental setup and ultrafast photon-induced tunneling. (a) Ultrashort ∼6 fs long laser pulses, central wavelength ∼ 810 nm, repetition rate ∼ 80 MHz, pulse energy ∼ 1–100 pJ, and intensity ∼ 10^12^ W/cm^2^ are focused at the apex of a W nanotip in tunneling contact with a Au(111) surface. (b) Illustrative representation showing the resonant dipole interaction between two electronic levels induced by ultrashort laser pulses at the tunnel junction of the STM. Here, the electronic level |1⟩ in the sample side of the junction has been vertically lifted above the Fermi level of the nanotip (*E*_F,t_). Tunneling from the two states (|1⟩and |2⟩) to the other side of the junction (|μ⟩ and |ν⟩) is denoted by the orange arrows.

## Results and Discussion

### Photon-Induced Tunneling Current

Before describing the different optical modulation techniques in the photon-driven regime, we analyze and describe below the origin and signatures of the photon-induced tunneling current at the STM junction.

The photon-driven tunneling regime is the weak-field regime, implying that incident photons gently perturb the system without distorting the potential energy landscape of the STM junction, which would be the case in the strong-field regime. Thus, the interaction of photons constituting an ultrashort laser pulse can be described within the framework of time-dependent perturbation theory.^28,29^ An incident photon (*ℏω*) interacts with the tip and sample states independently. A schematic illustration of the interaction of photons with a generic two level electronic system is shown in Figure 1b. We employ here the first-order perturbation theory,
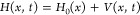
1where *H*_0_(*x*) is the unperturbed Hamiltonian describing the one-dimensional (*x*-axis is along the tip axis) STM junction and *V*(*x*,*t*) is the small time-dependent perturbation term.

We consider two independent Hamiltonians: in the tip (*H*^*Tip*^) and the sample side (*H*) of the junction.

*ϕ*_*n*_(*x*,*t*) are the eigenstates of the sample Hamiltonian;
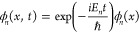
2Assuming Ψ(*x*,*t*) to be the approximate solution for the time-dependent Schrödinger equation (perturbed Hamiltonian), Ψ(*x*,*t*) can be expressed as a linear combination of the Eigen states of the unperturbed Hamiltonian.
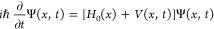
3
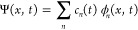
4Since *H*_0_ ϕ_*n*_^(0)^ = *E*_*n*_^(0)^ ϕ_*n*_^(0)^,
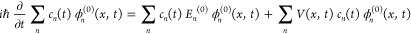
5Taking the inner product of eq 5 with ϕ_*m*_^(0)^(*x*,*t*);
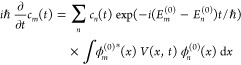
6Defining *V*_*mn*_(*t*) = ∫ ϕ_*m*_^(0)*^(*x*) *V*(*x*,*t*) ϕ_*n*_^(0)^(*x*) d*x* and , we get
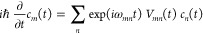
7Since  and the majority of the population remains in the initial state in the weak-field interaction,
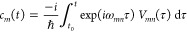
8with the initial condition of the system being *c*_*m*_(*t* = 0) and *c*_*n*_(*t* = 0) = 1.

Assuming a dipolar interaction of the laser pulses with the STM junction and for simplicity assuming that the laser pulses have a square shape (*G*(*t*)) with a central carrier frequency of ω,
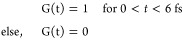
The temporal profile of the electric field of the laser pulse reads as
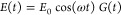
The interaction of electrons with the laser pulses within the dipole approximation can be expressed as
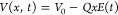
where *Q* is the net charge in the system.
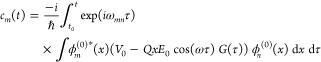
9Defining *M*_*mn*_ = ∫ ϕ_*m*_^(0)*^(*x*) *E*_0_*xQϕ*_*n*_^(0)^(*x*) d*x*,
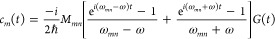
10Applying the rotating wave approximation,^30^
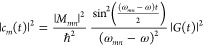
11The density of states (DOS) at the STM junction is not uniform like in an atom.^31^ |*c*_*m*_(*t*)| will be stronger in amplitude when the DOS is higher, implying that the above terms should be multiplied by the DOS of the initial state and the final state, *ρ*_*S*_(ε), integrated over the energy–width around the initial and final states that are coherently coupled by the laser pulse (Δ_Laser_); subindex *S* refers to the sample, and d*ε* refers to the integration variable over energy. Furthermore, the population of the electronic levels will also be weighted by the Fermi–Dirac population distribution, *f*_*S*_(ε).

Considering Δ_Laser_ to be the bandwidth of the exciting laser pulse,
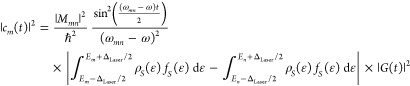
12Because the energies of electronic states of molecules (2–3 eV) are typically larger than the bandwidth of the laser pulses (∼0.4 eV), i.e., *E*_*m*_ ≫ Δ_Laser_, the above expression can be simplified to
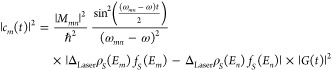
13The same term exists in the other side of the junction, i.e., in the tip side of the STM junction; superscript index Tip refers to the nanotip side of the STM junction.
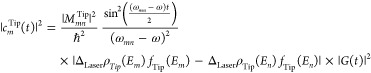
14The net tunneling current is proportional to the difference of the above two terms, with contributions from all of the involved electronic states in the system;
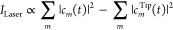
15The above expression of the tunneling current can be simplified for a two level system; please see Figure 1b. Here, the populations of the two states can be expressed as
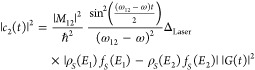
16
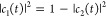
17
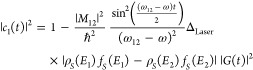
18where *M*_12_ = ∫ ϕ_1_^*^(*x*) *E*_0_*Qx* ϕ_2_(*x*) d*x*. *E*_1_ and *E*_2_ are the energies of the two electronic levels, and *ℏω*_12_ is the energy gap between them.

While performing time-dependent oscillations between the two dipole coupled states, electrons can tunnel to the other side of the barrier, hence contributing to the tunneling current. At any given time, the majority of the electronic population is in the ground state, because we are operating in the weak-field or perturbative regime of interaction.^24,28^ Here, the population of the ground state is higher than all of the other states i.e., *c*_1_ > *c*_2_, *ρ*_*S*_(*E*_1_) *f*_*S*_(*E*_1_) > *ρ*_*S*_(*E*_2_) *f*_*S*_(*E*_2_). Assuming that the DOS of the nanotip of the STM does not have any variation with energy and that the shape of its wave functions has an s-orbital distribution,^31^ the transition moment matrix element between the electronic states in the tip would be zero as per the Laporte selection rule, as it involves electronic states with identical parity (s-wave function). A zero transition moment matrix element would imply zero contribution of the states of the nanotip to the photon-induced tunneling current. Hence, the measured tunneling current will primarily be proportional to the population of the ground state, i.e., |*c*_1_(*t*)|^2^.
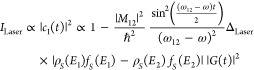
19
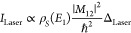
20when ω_12_ ≈ ω, i.e., when the incident photons are resonant with the two level system (Figure 1b).

For electron tunneling between a state ϕ_1_ or ϕ_2_ from the sample side to an aligned level on the tip side of the junction, *ϕ*_*μ*_ for ϕ_1_ and ϕ_ν_ for ϕ_2_ (see Figure 1b), one also has to consider the tunneling probability between these states. The tunneling probabilities between these states are given by the Bardeen’s tunneling matrix elements,^32^*M*_1μ_ and *M*_1ν_, respectively. Thus, eq 20 will be modified to account for the tunneling probabilities,
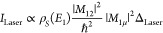
21when ω_12_ ≈ ω.

For the sake of simplicity we will ignore this term in our discussion in the following sections.

As can be seen from the above equations, the photon-induced tunneling current is proportional to the DOS of the initial state of the dipole interaction and the transition moment dipole matrix element (*M*_12_). The transition moment dipole matrix element determines the strength of the coupling of the initial state with the excited state. The initial state of the interaction of the laser pulses with the STM junction can be simply tuned by varying the DC bias at the junction^7^ as we show later. The line widths of electronic levels of molecules directly adsorbed on metallic surfaces or on an insulating film over metallic surfaces are usually very broad, >0.5 eV. The line width of an electronic level which can be coherently coupled by the laser pulses is determined by the bandwidth of the ultrashort laser pulse. In the present case the bandwidth of the laser pulses is ∼0.4 eV, implying that the entire electronic level of the molecule can be coherently excited by the laser pulses.

The above formulation for the ultrafast photon-induced tunneling current involves single-photon-induced transitions. In the case of multiphoton-induced transitions between electronic levels only the terms of the dipole matrix element, *M*_12_, have to be modified in the above formalism. For the case of two-photon transitions coupling the two states (ϕ_2_ and ϕ_1_), there will be a two-step transition, first from the initial state ϕ_1_(*E*_1_) to a virtual level *ϕ*_*ν*_ and then from *ϕ*_*ν*_(*E*_*v*_) to the final state ϕ_2_(*E*_2_). The second-order transition moment matrix element in this case can be expressed as
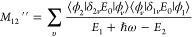
22where ℏω is the energy of the photon absorbed in the first transition, δ_1ν_ and δ_2ν_ are the dipole moments, and *E*_0_ is the peak field strength of the laser pulse inducing transitions. Here we assume that the photon energies of the two absorbed photons are identical, ℏω. A similar expression as shown in eq 22 can be derived for three and higher order photon transitions. The presence of single-photon- and multiphoton-induced transitions on integration of ultrashort laser pulses in an STM junction has been demonstrated recently.^8,19^

### Dispersion-Modulated Ultrafast Tunneling Microscopy

Even though photon-induced tunneling lies in the weak-field interaction of the laser pulses with the STM junction, the tunneling current is still proportional to the peak strength of the laser pulses as would be the case in the strong-field interaction. A recent work has theoretically evaluated the contribution of different photon orders in the photon-induced tunneling current at the STM junction; the tunneling current has been shown to scale with respect to the order of the peak incident electric field strength for various photon-order processes.^33^ Two laser pulses with identical pulse energies but different peak intensities will generate different amplitudes of photon-induced tunneling current. For example, a laser pulse of 10 fs duration will generate a higher tunneling current compared to a laser pulse of 15 fs duration, even though their pulse energies are identical, e.g., 50 pJ. It is crucial that the energies of both laser pulses are the same to maintain a constant heat load at the STM junction.

Here, we introduce a technique of dispersion modulation at the STM junction. Dispersion of the laser pulses is modulated between *low* “short pulse” and *high* “long pulse” at frequencies as small as 1 kHz. High dispersion implies longer pulse duration, and low dispersion implies shorter pulse duration, i.e., higher peak field strength. A shorter pulse with higher peak field strength would generate higher nonlinear tunneling current (arising from multiphoton processes) compared to a longer pulse of lower peak field strength. The difference in the tunneling current induced by the two pulses is detected by lock-in measurement at the frequency of the dispersion modulation.

**Figure 2:**
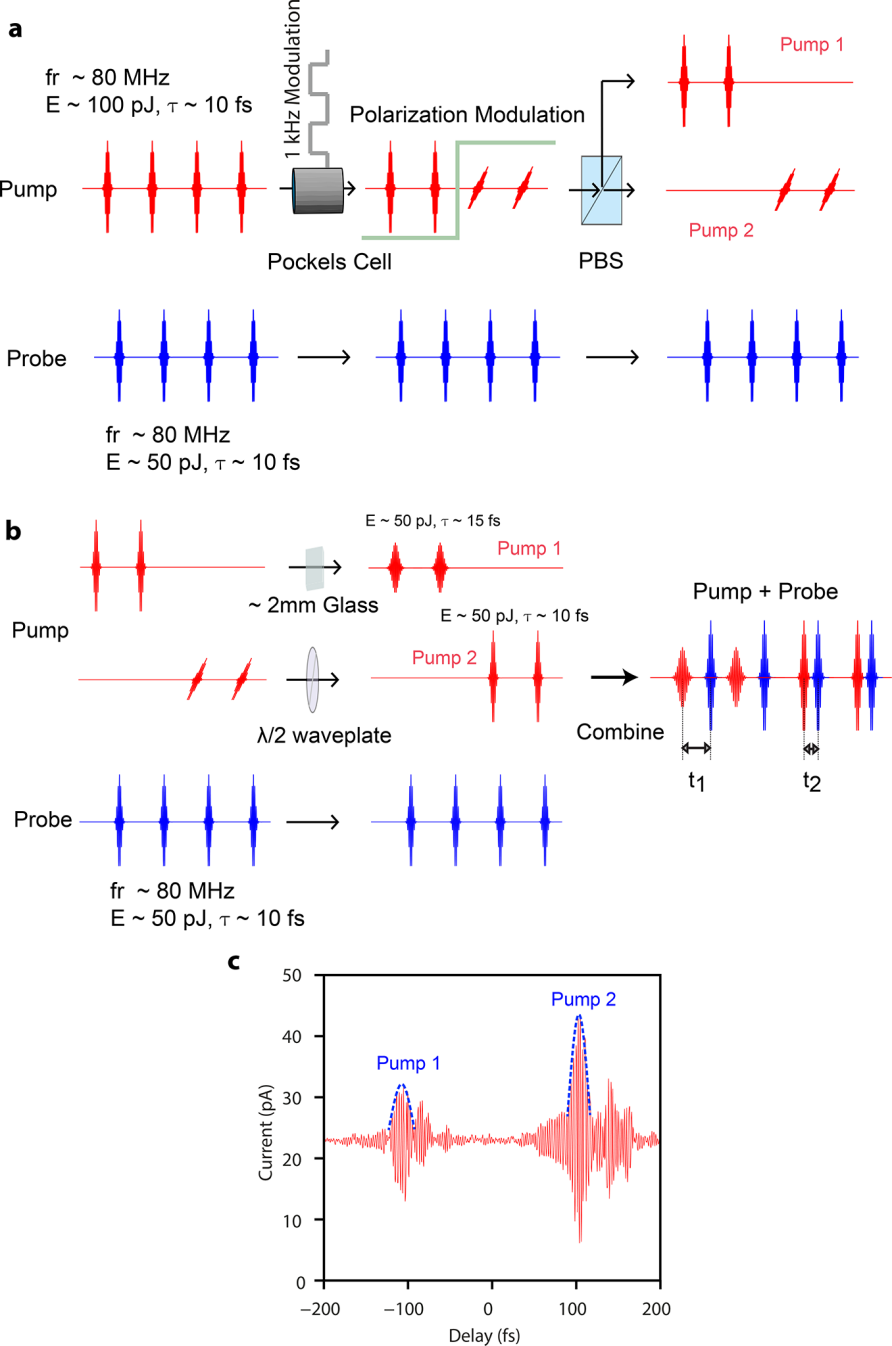
Time-resolved dispersion modulated tunneling microscopy. (a) Linearly polarized ultrashort pump laser pulses (red) coming at a repetition rate of ∼80 MHz with their polarization modulated between s (vertical) and p (horizontal) at a frequency of ∼1 kHz after traversing through a Pockels cell operating at that frequency. The s- and p-polarized laser pulses are separated by a polarizing beam splitter (PBS). (b) The s-polarized beam passes through a ∼2 mm thick fused silica glass stretching the pulse duration to ∼15 fs; whereas the p-polarized laser pulses traverse through a thin half-waveplate (λ/2) rotating their polarization from p to s, the duration of the laser pulses in this arm is ∼10 fs. The laser pulses from the two pump arms (pump 1 and pump 2) are then combined with the probe pulses (blue) and focused at the STM junction. (c) Measured variation of the laser-induced tunneling current when the delay of the probe pulses (blue) is swept across Pump 1 and Pump 2 pulses. The variation of the laser-induced tunneling current in panel c was measured in the constant current mode of the STM, *I*_set_ = 100 pA, and DC bias = 1 V. The measurements were performed with an STM junction comprising a W nanotip and a Au(111) surface.

Ultrashort laser pulses (red pulses in Figure 2a) traversing through a Pockels cell have their polarization modulated between horizontal (p) and vertical (s) at the driving frequency of the Pockels cell (∼1 kHz). The laser pulses are precompensated for their dispersion accumulated by traversing through a ∼2 cm thick potassium dideuterium phosphate crystal (Pockels cell) by 10 bounces off a pair of chirped dielectric mirrors with a group delay dispersion of ∼−120 fs^2^. The two orthogonally polarized pulse trains (red pulses in Figure 2a) are separated into two different arms by a polarizing beam splitter (PBS). The vertically polarized pulses are temporally dispersed by a ∼2 mm thick fused silica glass, whereas the horizontally polarized laser pulses are rotated back to vertical polarization by a half-wave plate, as shown in the top panel of Figure 2b (red pulses). The two pulses (Pump 1 and Pump 2) as shown in Figure 2b have pulse durations of ∼15 and 10 fs, respectively, each with a pulse energy of ∼50 pJ. After combination of the two pulse trains (Figure 2b), the laser pulses at a repetition rate of ∼80 MHz are modulated in their pulse duration from short (∼10 fs) and long (∼15 fs) at a frequency of ∼1 kHz.

This dispersion modulated pump pulse train (red) is then combined with a probe pulse train (blue pulses in Figure 2b). The pulses in the probe train come at the same repetition rate as the pump pulses, with their duration being ∼10 fs and a pulse energy of ∼50 pJ. The probe pulses have different delays with respect to the two pump pulses (*t*_1_ and *t*_2_); this delay can be independently controlled and stabilized. The variation of the laser-induced tunneling current when the delay of the probe pulses is swept across the two pump pulses is shown in Figure 2c. An autocorrelation of the laser-induced tunneling current between pump 1 and probe pulse as well as pump 2 and probe pulse can be clearly seen. The laser-induced tunneling current is higher for the pump 2 pulse compared to the pump 1 pulse owing to its shorter pulse duration.

It is important to note here that it is not the tunneling current induced by pump 1 and pump 2 pulses that we are measuring but rather the difference in the tunneling current induced by the two pulse trains individually, which are modulated at a small frequency of ∼1 kHz. Also, it is not the same probe pulse which overlaps with the two pump pulses; these probe pulses are separated by 11 ns. In absolute terms, when the probe beam has a delay of *t*_1_, e.g., 100 fs from the pump 1 beam, the delay of the pump 2 beam with respect to the probe beam is 11 ns + 100 fs or a multiple of 11 ns + 100 fs. Since the signal from both of the pairs of pump–probe delays comes at the same frequency in the lock-in detection, i.e., driving frequency of the Pockels cell, the laser-induced tunneling current signal will appear only with relative delays between the two pump and probe pulses.

The technique of dispersion modulation can be used to perform pump–probe measurements on single molecules adsorbed on metallic surfaces. Here one of the pulses, pump 1 or pump 2 can be sufficiently separated from the probe pulse so as not to interfere in probing of the dynamics. By varying the delay between the selected pump and the probe pulses, the dynamics in the system can be probed as in a conventional pump–probe experiment.

In order to ascertain that the presented dispersion modulation technique preserves the key characteristics of an STM, i.e., exponential dependence of the tunneling current on the tunnel gap width and atomic spatial resolving capability, we have performed characterization measurements on a 1:1 heteromolecular assembly of tetrathiafulvalene (TTF) and tetracyanoquinodimethane (TCNQ) molecules on a Au(111) surface. Here we consider the scheme of a 80 MHz pulse train, where the dispersion of the pulses is modulated at a slow frequency of ∼1 kHz, as shown in Figure 2a. The laser-induced tunneling current as detected by lock-in measurement is the difference in the tunneling current induced by the short and long pulses, at the frequency of ∼1 kHz.

**Figure 3:**
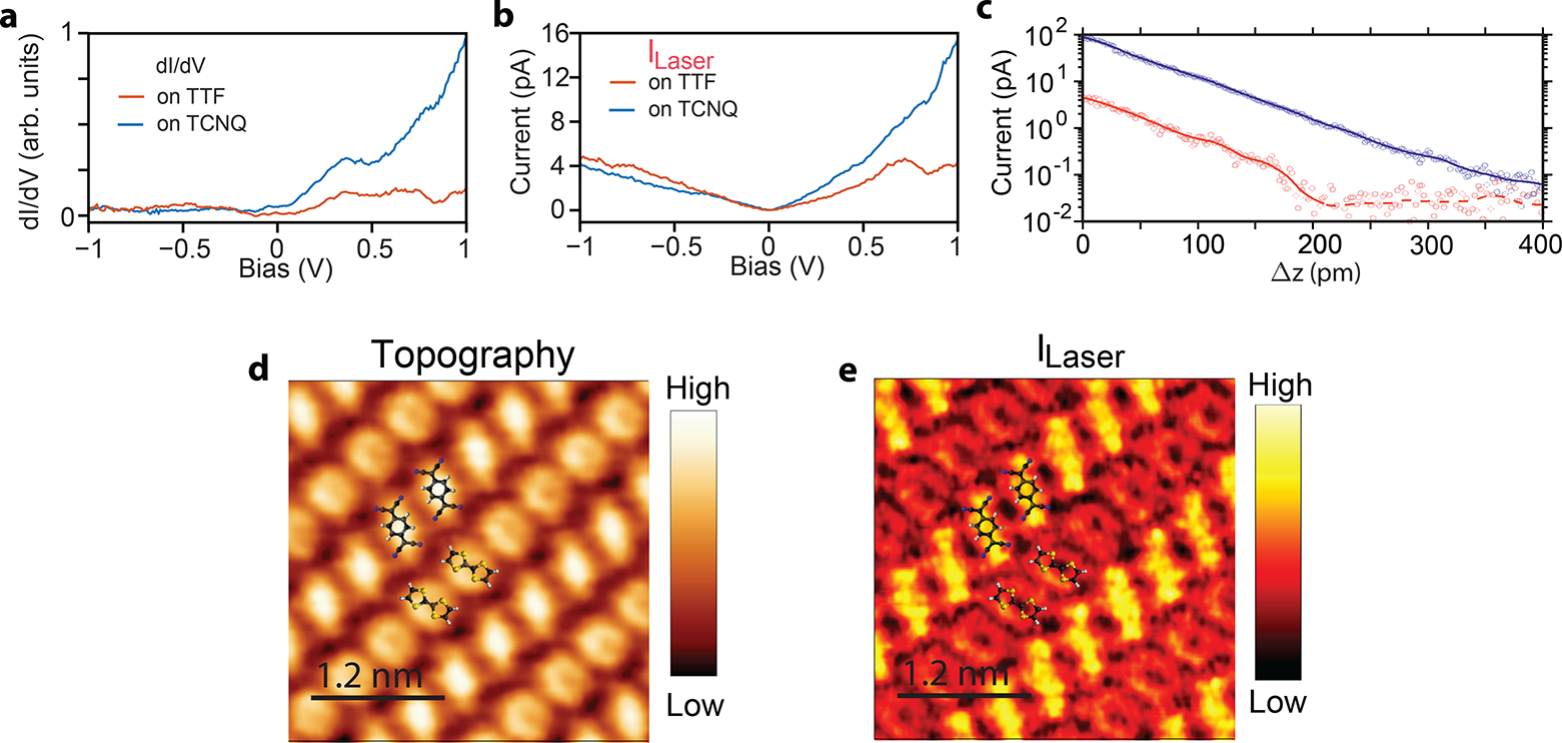
Time-resolved dispersion modulated tunneling microscopy. (a) Differential conductance (d*I*/d*V*) measurement of a tetrathiafulvalene (TTF, red curve) and a tetracyanoquinodimethane (TCNQ, blue curve) molecule constituting of a 1:1 heteromolecular assembly on a Au(111) surface. (b) Variation of the laser-induced tunneling current as a function of the DC bias measured on a TTF (red curve) and TCNQ (blue curve) molecule. (c) Comparison of the variation of the DC tunneling (blue curve) and laser-induced tunneling (red curve) currents for increasing tunneling gap widths. The dashed part of the red curve represents the noise floor in the measurement. DC bias of 1 V and set current of 200 pA were used. (d, e) Simultaneously recorded variation of the *z*-topography (d) and the laser-induced tunneling current (e) for a 1:1 TTF-TCNQ complex on Au(111). Steady state STM imaging was performed in the constant current mode with a set point of 200 pA and a DC bias of 0.25 V. Scaled ball and stick models of TTF and TCNQ molecules have been superimposed as a guide to the eyes.

A differential conductance (d*I*/d*V*) measurement on top of a TTF and a TCNQ molecule is shown in Figure 3a. The simultaneously recorded variation of the laser-induced tunneling current as a function of the DC bias (*I*_Laser_ vs *V*) is shown in Figure 3b. The laser-induced tunneling current maximizes when the initial state of the photon-driven interaction is aligned to the electronic level of the system, as in a differential conductance measurement. d*I*/d*V* curves throughout this work were measured by lock-in detection by applying an AC modulation bias with an amplitude of 20 mV at a frequency of 887 Hz.

The dependence of the DC and photon-induced tunneling currents on increasing tunnel gap width (*I*–*z* curve) is shown in Figure 3c. The *I*–*z* curve was measured on top of a Au(111) surface with a DC bias of 1 V and set current of 200 pA. Comparison of *I*_DC_ vs *z* and *I*_Laser_ vs *z* curves shows that the photon-induced tunneling current has a similar exponential dependence on the tunnel gap width as for the case of the DC tunneling current.

The spatially resolved variation of the photon-induced tunneling current for a 1:1 heteromolecular assembly of TTF and TCNQ molecules in the constant current mode of the STM is shown in Figure 3e. The simultaneously recorded *z*-topography is shown in Figure 3d. TTF molecules display a ring-like spatial distribution, whereas TCNQ molecules have an elongated shape also displaying submolecular features. The laser-induced tunneling current is higher for the TCNQ molecules compared to the TTF molecules.

All measurements shown in Figure 3 and Figure 2 attest to the fact that this technique of dispersion modulation of laser pulses at the STM junction enables atomic spatial resolving capability along with sub-femtosecond temporal resolution.

### Polarization-Modulated Ultrafast Tunneling Microscopy

The spatial distribution of the electric field of incident laser pulses at an STM junction is substantially affected by the broken spatial symmetry of the junction. Strong geometrical confinement of the laser pulses in the nanocavity of the STM junction leads to a significantly higher peak field strength compared to the free-space peak field strength.

**Figure 4:**
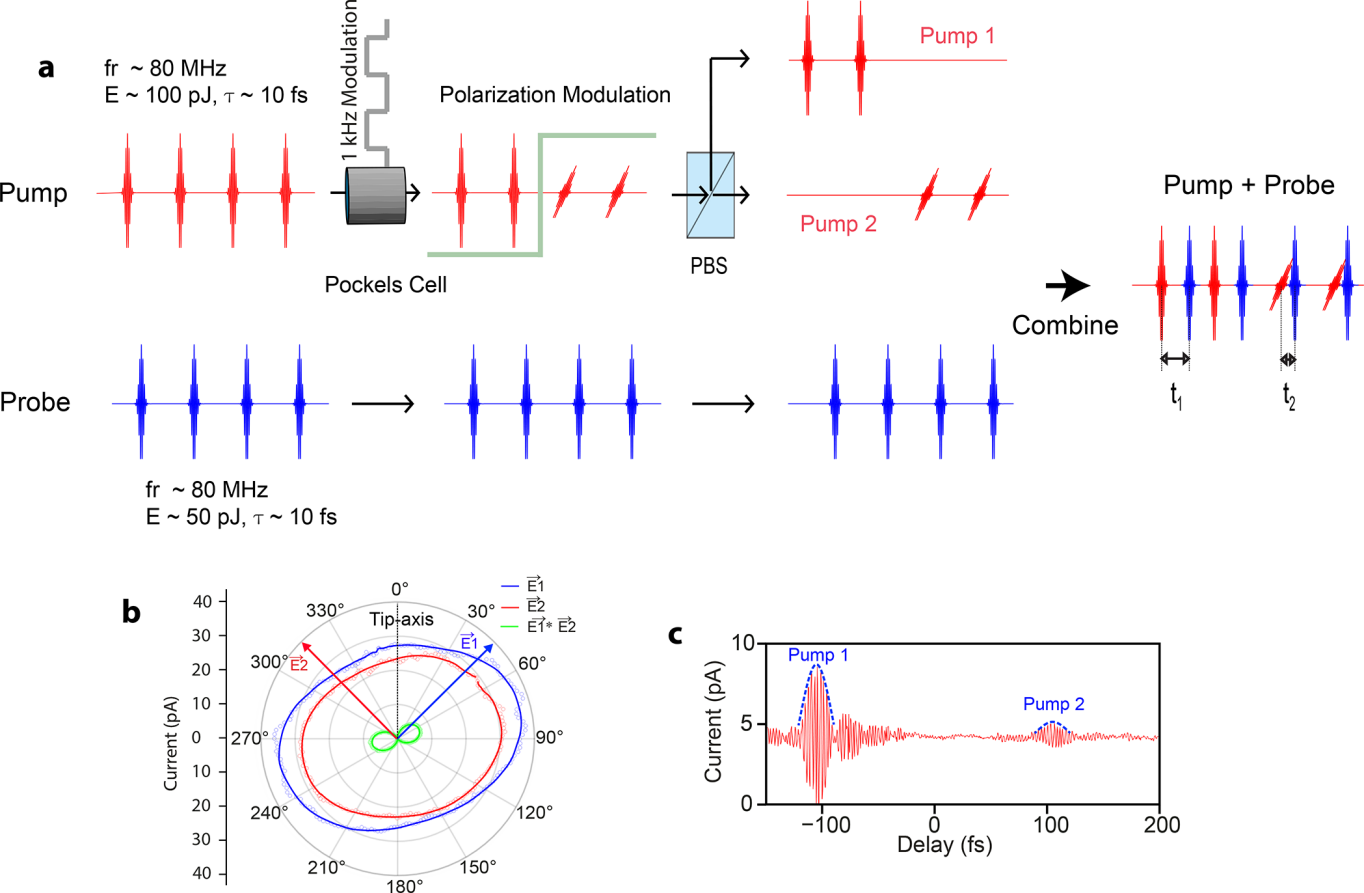
Time-resolved polarization modulated tunneling microscopy. (a) Ultrashort laser pulses have their polarization modulated at ∼1 kHz frequency after traversing through a Pockels cell. The two orthogonally polarized trains of s and p laser pulses (pump 1 and pump 2) are separated by a polarizing beam splitter (PBS). The train of polarization modulated pump 1 and pump 2 pulses is then combined with s-polarized probe pulses (blue), which come at a repetition rate of ∼80 MHz. The combined pulse trains are then focused at the STM junction. (b) Measured variation of laser-induced tunneling current as a function of rotating the polarization of pump 1 (blue-curve) and pump 2 (red-curve) pulse trains and when the two pulse trains are concurrently incident (green curve) on the STM junction. (c) Measured variation of the laser-induced tunneling current when the delay of the probe pulses (blue) is varied across the pump 1 and pump 2 pulses. The variation of the laser-induced tunneling current in panel c was measured in the constant current mode with *I*_set_ = 100 pA and DC bias = −1.8 V with a W nanotip on a Au(111) sample.

High-repetition-rate laser pulses (∼80 MHz) traversing through a Pockels cell have their polarization modulated between vertical (s) and horizontal (p) at the driving frequency of the Pockels cell (∼1 kHz). The two orthogonally polarized pulse trains are annotated as pump 1 and pump 2 in Figure 4a. The variation of the photon-induced tunneling current as a function of the polarizations of the pump 1 and pump 2 pulses across the tip axis (0°) is shown in Figure 4b. The measurement has been performed on a perylenetetracarboxylic dianhydride (PTCDA) molecule constituting a monolayer coverage on top of a Au(111) surface with a W tip. Here, while performing the measurement for the pump 1 pulse train, the pump 2 pulse train is not present and vice versa. A very uniform distribution of the photon-induced tunneling current with variation of <20% is measured for the two polarizations of the laser pulses. The small nonhomogeneity of the photon-induced tunneling current only arises due to the spatial asymmetry of the transition moment matrix elements. When both pump 1 and pump 2 pulse trains are incident at the STM junction, we measure the difference of the photon-induced tunneling current induced by the two pulses as shown by the green curve in Figure 4b. A mathematical difference of the red and blue curves produced by the polarization dependence of the pump 1 and pump 2 pulse trains matches the green curve which is generated when both of the pulse trains are incident at the STM junction. This observation connotes the fact that the far-field orthogonality of pump 1 and pump 2 pulse trains is preserved in the near field of the STM junction. In the case of near-field space–time dipolar coupling between the pump 1 and pump 2 pulses, we would measure a more complex variation of the laser-induced tunneling current which would not be the simple difference induced by both pulse trains as in Figure 4b.

The observation of a uniform photon-induced tunneling current on change of polarization of the laser pulses with respect to the nanotip axis is quite striking. Even for a tungsten tip (nonplasmonic) the field enhancements are different along the nanotip axis and perpendicular to it, due to the lightning rod effect. The photon-induced tunneling current is proportional to the DOS of the initial state and the transition moment dipole matrix element coupling the initial state of interaction with other excited states. The transition moment matrix element for a single-photon transition is given as
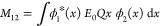
23where *Q* is the net charge in the system, ϕ_1_ and ϕ_2_ are two electronic states dipole coupled by a laser pulse of peak field strength of *E*_0_.
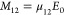
24where μ_12_ is the dipole matrix element. The transition moment matrix elements for the parallel and perpendicular components of the incident laser pulse with respect to the molecular axis can be expressed as
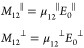
25The nanotip axis (*E*_0_^⊥^) is perpendicular to the molecular axis (*E*_0_^∥^).

Even though *E*_0_^⊥^ is larger than *E*_0_^∥^, due to the lightning rod effect, the dipole matrix elements parallel to the molecular axis (perpendicular to the nanotip axis) μ_12_^∥^ are larger than the ones along the nanotip axis μ_12_^⊥^ by several factors.^7^ For a monolayer of PTCDA molecules adsorbed on a Au(111) surface, μ_12_^∥^ > μ_12_^⊥^ by a factor of nearly 8. Owing to this fact, the transition moment matrix elements for the parallel and perpendicular orientations of the laser pulse with respect to the nanotip axis will become comparable in the current case.
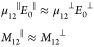
26This is the reason behind a very uniform variation of the photon-induced tunneling current on change of the polarization of the laser pulses at the STM junction for a nonplasmonic nanotip.

Orthogonal pump 1 and pump 2 pulse trains can be combined with a probe pulse train, as shown by the red and blue pulses in Figure 4a. This combination produces orthogonal pump–probe pulse trains, with the delay between the pump–probe pulses denoted by *t*_1_ and *t*_2_, respectively. An autocorrelation between pump 1 and pump 2 pulses with the probe pulse is shown in Figure 4c. The delay between pump 1 and pump 2 pulses can be randomly set. This technique of polarization modulation can be used to perform pump–probe measurements, provided that the delay of either pump 1 or pump 2 pulses is significantly far from the probe pulse, as described in the previous section of dispersion modulation.

We should note that metallic nanotips for which the plasmonic resonances fall within the bandwidth of the laser pulse, such as Ag or Au tips, display a much larger field enhancement along the tip axis compared to that orthogonal to it, owing to the excitation of localized surface plasmon resonances.^34^ In the case of plasmonic nanotips the photon-induced tunneling current will not have a uniform dependence upon rotation of the polarization of the laser pulse along the tip axis, but rather a skewed dependence. The photon-induced tunneling current will be much higher along the tip axis. Moreover, the far-field orthogonality of pump 1 and pump 2 pulse trains will not be preserved in the near-field of the STM junction.

### Frequency-Modulated Ultrafast Tunneling Microscopy

**Figure 5:**
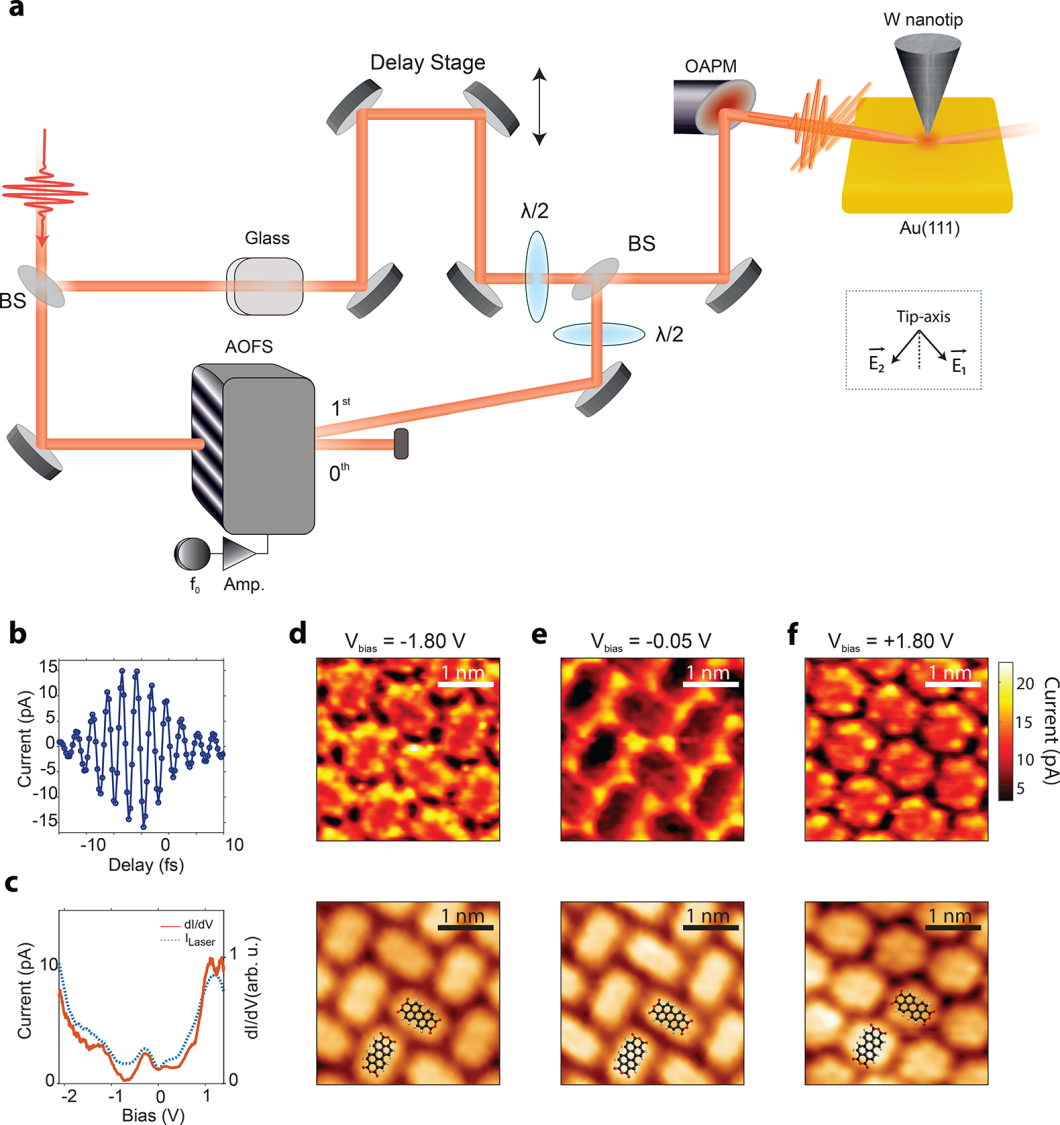
Time-resolved frequency modulated tunneling microscopy. (a) The carrier frequency of ultrashort laser pulses is slightly shifted by passing them through an acousto-optic frequency shifter (AOFS). The polarization induced at the STM junction by the frequency shifted first-order diffracted beam from the AOFS and the unshifted beam interfere at the small frequency offset between the two pulses. The two laser pulses are orthogonally polarized with respect to each other and form an angle of 45° with respect to the axis of the W nanotip as shown in the inset. (b) Variation of the laser-induced tunneling current as a function of the delay between the two pulses. DC bias of −1 V and set current of 100 pA were used. (c) The DC differential conductance (solid orange-curve) and the variation of the photon-induced tunneling current (dashed blue curve) are compared as a function of the DC bias at the STM junction for a PTCDA molecule on top of Au(111). (d–f) Spatially resolved variation of the laser-induced tunneling current at various biases at the STM junction, −1.8 V, −50 mV, and 1.4 V, respectively (top panels), and (bottom panels) *z*-topography at the same DC biases; the set point current for all images is 100 pA. BS, beam splitter; λ/2, half-waveplate; OAPM, off-axis parabolic mirror.

In this section, we will describe the technique of frequency modulation of laser pulses to lock-in-detect the laser-induced tunneling current, which was originally proposed by the group of Prof. Apkarian and recently advanced by us.^7,13^ The carrier frequency of laser pulses can be slighted shifted (<1 kHz) by focusing the laser pulses to an acousto-optic frequency shifter (AOFS). The first-order diffracted laser beam out of the AOFS will be upshifted in its carrier frequency by the frequency at which the AOFS is driven, whereas the zeroth-order diffracted beam will be unaffected. In our experiments, laser pulses coming at a repetition rate of ∼80 MHz are loosely focused to an AOFS, which is driven at the frequency of 80 MHz + 1 kHz as shown in Figure 5a. The photon-induced tunneling current can be lock-in-detected by homodyne beating of the polarization induced by frequency shifted and unshifted laser pulses at the STM junction.

An ultrashort carrier-envelope-phase (CEP) stable laser pulse is comprised of an underlying frequency comb, whose teeth are separated by the repetition rate (*f*_*r*_ ∼ 80 MHz) of the laser. This frequency comb spans the entire spectral bandwidth of the laser pulses, i.e. from 650 to 1050 nm, with the central wavelength of the spectrum being at 810 nm. The electric field of one of the laser pulses (zeroth order) in the frequency domain can be written as
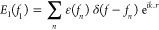
27where *f*_*n*_ = *nf*_*r*_, is the *n*th multiple of the repetition rate of the laser pulses. δ(*f* – *f*_*n*_) is the Dirac δ function describing the frequency comb separated by the repetition rate of the laser pulses and ε(*f*_*n*_) describes the spectral weight of each individual comb line in the laser pulse. The central frequency (*f*_1_) of our laser pulses is 0.37 × 10^15^ Hz (for a central wavelength of λ ∼ 810 nm), implying that *n* ∼  is approximately 4.6 × 10^6^. The electric field of the second laser pulse, first-order diffracted beam out of the AOFS, which is upshifted in the frequency by *f*_*r*_ + *f*_0_ with respect to the first pulse (zeroth order) can be written as
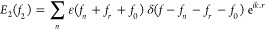
28
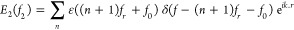
29Since the order of the frequency comb lines *n* is much greater than 1 (*n* ∼ 4.6 × 10^6^), implying (*n* + 1) ∼ *n*, the above equation simplifies to
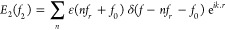
30
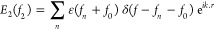
31Thus, the carrier frequency of the two laser pulses (zeroth and first order) is separated by a small offset frequency of *f*_0_ (<1 kHz). In our experiment, the first-order diffracted beam is combined with the laser beam which does not traverse through the AOFS at the STM junction. Laser pulses not traversing through the AOFS are identical in its carrier frequency compared to the zeroth-order diffracted beam. The two laser pulses are orthogonally polarized with respect to each other and form an angle of 45° with respect to the nanotip axis, as shown in the inset of Figure 5a. The net electric field of the two laser pulses at the STM junction at a relative delay of τ between them can be expressed as
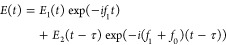
32The electric field will have components both along the tip axis as well as perpendicular to it.

Assuming a single-photon-induced transition dipole coupling two electronic states (ϕ_1_ and ϕ_2_, Figure 1b), the photon-induced tunneling current is proportional to the square of the transition moment matrix element (eq 20);
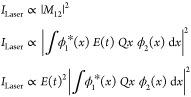
33Thus, the tunneling current at a delay τ, between the two laser pulses, can be expressed as
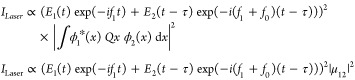
34where *E*_1_(*t*) and *E*_2_(*t*) are the intensity envelopes of the two laser pulses, with their carrier frequencies being *f*_1_ and *f*_1_ + *f*_0_; τ is the delay between the two pulses. μ_12_ is the dipole matrix element between the states ϕ_1_ and ϕ_2_.
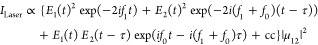
35The nonlinear polarization induced by the laser pulses at the STM junction is the origin of the photon-induced tunneling current. In the above equation most of the terms contain very high frequencies such as 2*f*_1_ and 2(*f*_1_ + *f*_0_). The tunneling current generated by these polarization terms cannot be lock-in detected. The photon-induced tunneling current at such high frequencies is mixed with the DC tunneling current at 0 Hz. However, there is a polarization term arising due to homodyne beating between the two laser pulses, which comes at the small offset frequency of *f*_0_ (<1 kHz); this frequency falls within the bandwidth of the high gain amplifier used in the STM.
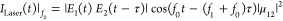
36The amplitude of the two pulses in the experiment is equal, simplifying the above equation as
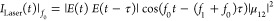
37A correlation of the photon-induced tunneling current as a function of the delay between the two pulses is shown in Figure 5b. This correlation has been measured on a clean Au(111) surface, with a DC bias of −1 V and a DC set current of 100 pA. The periodic oscillation in the tunneling current in Figure 5b is related to the phase term of the argument of the cosine function in eq 37, which varies as (*f*_1_ + *f*_0_)τ, i.e., the carrier frequency of the laser pulses. At zero delay between the two pulses (τ = 0), the nonlinear polarization in eq 37 imitates the response of the polarization induced by laser pulses coming at the small repetition rate frequency of *f*_0_. Here, there will only be an electric field component along the tip axis.
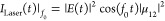
38A similar expression for the photon-induced tunneling current can be derived for the two-photon and three-photon tunneling processes by replacing *M*_12_ in eq 33 by *M*_12_″ and *M*_12_‴, i.e., second- and third-order transition matrix elements (eq 22). It is important to note that several scaling experiments^8,19^ have shown that the number of photons involved in the photon-induced tunneling process is dominated by a mixture of different photon-order processes and not by just a single photon-order process.

The key benefit of using this frequency modulation technique at the STM junction is that one can imitate the laser interaction of a single pulse, without the need of mechanical modulation of the laser pulses. However, it is important to note that the delay between the two arms of the interferometer should not change over time; the required deviation in the interferometric stability is <100 attoseconds (as) for several hours.

A differential conductance measurement of a single PTCDA molecule constituting a monolayer on top of Au(111) is shown in Figure 5c (solid orange curve). The corresponding variation of the photon-induced tunneling current for a single-pulse scenario (eq 38, τ = 0 fs) as a function of the DC bias is shown in Figure 5c (dashed blue curve). The local maxima of the photon-induced tunneling current overlap with the positions of the local maxima in the differential conductance measurement. This observation is in agreement with the equations presented in the “photon-induced tunneling current” section, validating the photon-induced dipole coupling picture as the source of the measured laser-induced tunneling current. A differential conductance measurement on top of a clean Au(111) surface depicting the presence of its surface state at ∼−0.5 V is shown in Supporting Information (SI) Figure S1.

The spatial variation of the photon-induced tunneling current for a single laser pulse (eq 38, τ = 0 fs) for a monolayer of PTCDA molecules on top of Au(111) at different biases is shown in the top panels of Figure 5d–f; the simultaneously recorded *z*-topography images at the same biases are shown in the bottom panels. The spatial maps have been measured in the constant current mode of the STM with a set current of 100 pA. At DC biases of −1.8 and +1.8 V, photon-current maps resemble the spatial profile of highest occupied and lowest unoccupied molecular orbitals (HOMO, LUMO) of the PTCDA molecules. At a DC bias of −0.05 V, the photon-induced tunneling current is much higher on the interstitial sites between the molecules compared to the interior of the molecules itself as shown in Figure 5e (top panel). The *z*-topography in Figure 5e (bottom panel), nevertheless, implies that the DC tunneling current is higher on the molecules compared to the interstitial sites. This distinct observation stems from the fact that here the photon-induced tunneling current arises from the surface state of Au(111) and not from any molecular state of the PTCDA molecules.^35^ Moreover, the photon-induced tunneling current is directly proportional to the DOS of the initial state of the laser interaction and the corresponding transition moment matrix element.

In order to comprehensively contrast the capabilities of the different techniques introduced in this work, characterization measurements performed with the techniques of frequency and dispersion modulation for the molecular system of a monolayer of PTCDA molecules on Au(111) are compared in Figures S2 and S3 in the SI. A compelling similarity of the different measurements from the two different techniques can be clearly seen.

### Amplitude-Modulated Ultrafast Tunneling Microscopy

Amplitude modulation of laser pulses to lock-in detect small laser-induced tunneling currents is a very common approach. However, such modulation could be detrimental in an STM, wherein periodic modulation of laser pulses can lead to a periodic expansion and contraction of the nanotip of the STM. Nevertheless, as we have recently shown, the thermal expansion and contraction of nanotips is sensitive to the modulation frequency of the laser pulses, which can be used to minimize the effect.^8^

In the following we describe an approach to characterize the magnitude of the thermal expansion of the nanotip for varying modulation frequencies of the laser pulses.

**Figure 6:**
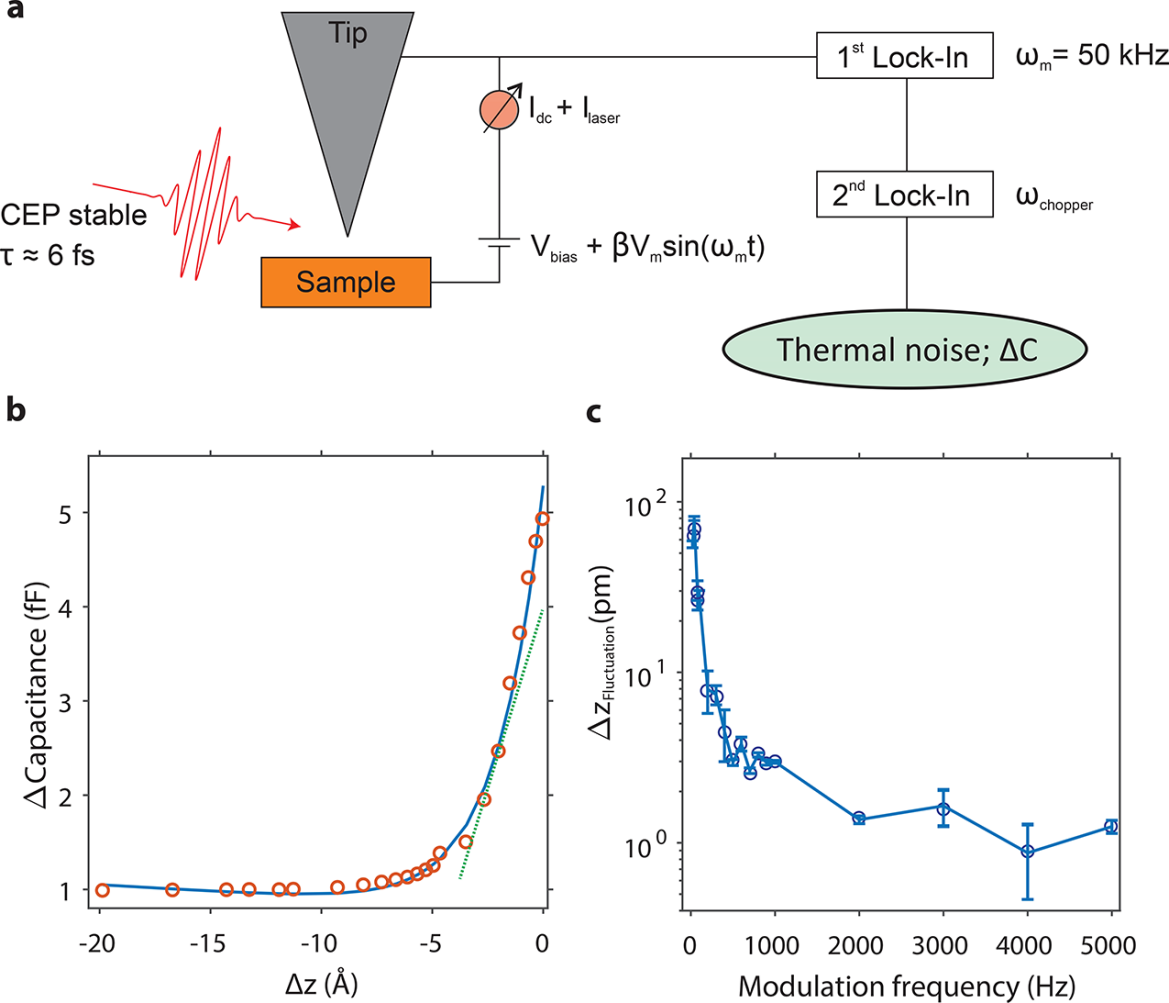
Thermal noise induced by amplitude modulation of laser pulses. (a) The schematic shows the experimental setup. (b) Relative change of the capacitance at the STM junction (W nanotip and Au(111) surface) is measured as a function of reducing tunnel gap width. The capacitance is measured at the frequency of the sinusoidal modulation voltage, *V*_*m*_ sin(ω_*m*_*t*). (c) Laser-induced thermal fluctuation of the tunneling gap width (Δ*z*) is shown for increasing amplitude modulation frequencies of the laser pulses.

An STM junction comprising a metallic tip and sample acts like a capacitor. The capacitance at the junction shows classical behavior in the nontunneling regime (). However, in the tunneling regime the capacitance depends exponentially on the tunneling gap width,^36^*C*(Δ*z*) ∝ exp(−ζΔ*z*). This extremely nonlinear behavior of the capacitance at the STM junction is an ideal observable to monitor minute changes at the tunneling gap width in real time. To quantitatively measure thermal effects, i.e., the periodic modulation of the length of the nanotip upon laser illumination, we apply a sinusoidal voltage in addition to the DC bias at a frequency that is much higher than the feedback bandwidth of the STM (∼500 Hz); *V* = *V*_0_ + *V*_*m*_ sin(*ω*_*m*_*t*), as shown in Figure 6a. The high amplification gain of 10^9^ V/A in the STM as used in the present case limits the operational bandwidth from DC to 100 kHz. The dependence of the capacitance on reducing the tunneling gap width is measured by lock-in detection at the frequency of the applied sinusoidal voltage, *ω*_*m*_ (50 kHz), first lock-in Figure 6a. The time constant in the first lock-in is 10 μs with the slope of low-pass-frequency filtering set at 24 dB, making an effective single-shot measurement time of 100 μs. The fast time scale of 100 μs at the first lock-in makes it sufficiently sensitive to any slower variation at the tunneling junction that comes from the laser modulation. The measured relative change of the capacitance is shown in Figure 6b. The output of the first lock-in is fed to a second lock-in detection (Figure 6a), the reference frequency for this lock-in being the modulation frequency of the laser pulses (ω_c_). The time constant in this lock-in measurement is slow enough (1 s) to measure a stable signal at the modulation frequency of the laser pulses.

Fluctuations of the tunneling gap width during laser illumination due to thermal effects come de facto at a frequency identical to the modulation frequency of the laser. This behavior arising from thermal effects, i.e., tunneling gap-width modulation, makes it difficult to unravel it from the real signal generated by the laser pulses at the STM junction, which also comes at the same frequency of the laser modulation. Here, we use an approach to measure the variation of the tunneling gap width during laser modulation. A variation of the tunneling gap width (Δ*z*) modulates the capacitance at the junction; for laser modulation at frequencies less than 10 kHz (ω_c_) a sinusoidal additional voltage around 50 kHz on the bias voltage is seen as a static bias. The temporal modulation of Δ*z* generates a current observable in the second lock-in measurement as
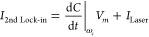
39
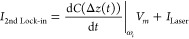
40Here, the first term arises purely from the thermal effects, whereas the second term is the real laser-induced tunneling current. An increase in the modulation voltage, *V*_*m*_, increases the contribution of the first term in the last equation by a factor (β) proportional to the voltage increase, leaving the second term unaffected that comes purely due to laser-induced tunneling.
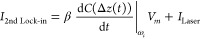
41Thermal fluctuations (Δ*z*(*t*)) of the tunneling gap width can then be found by subtracting eq 41 from eq 40
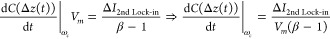
42
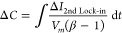
43Conversion of Δ*C* to Δ*z*_Fluctuation_ is performed by a calibration measurement of the capacitance as shown in Figure 6a at *ω*_*m*_ frequency with the effective voltage on the sample being *V* = *V*_0_ + *V*_*m*_ sin(*ω*_*m*_*t*). A depiction of the measurement procedure is shown in Figure 6a. The modulation (fluctuation) of the tunneling gap width due to thermal effects of the laser pulses at the STM junction for varying modulation frequency of the laser pulses is shown in Figure 6c. In order to attain maximum sensitivity in the fluctuation measurement of the tunnel gap, the region of maximum nonlinear change in the capacitance shown by the dashed green curve in Figure 6b is used in the experiment.

Modulation frequencies higher than 1 kHz lead to negligible thermal fluctuation (<1 pm) of the tunnel gap width with a pulse energy of 2.5 nJ as used in this experiment. Thus, low-energy ultrashort optical near-infrared pulses can be coupled into the tunneling junction without noticeable thermal noise, provided their amplitude modulation frequencies are sufficiently high (>1 kHz).

## Conclusion

Ultrafast photon-driven tunneling microscopy as accessed by the different optical techniques described in the current work can simultaneously achieve angstrom-scale spatial resolution and sub-femtosecond temporal resolution. We have analyzed the basics of how photons constituting an ultrashort pulse interact with an STM junction and how this interaction can be used to develop techniques capable of imaging electron dynamics at the atomic spatiotemporal limits. The variation of the photon-induced tunneling current as a function of the DC bias at the junction maps the electronic DOS of the system.

Dispersion and polarization modulation techniques enable lock-in detection of photon-induced tunneling currents by measuring the difference in the tunneling current generated by short and long pulses, and between two orthogonally polarized laser pulse trains, respectively. These two techniques do not require to mechanically modulate the laser pulses at the STM junction, hence maintaining a constant heat load at the junction. The laser pulses in the current work are separated by ∼11 ns, which is much shorter than the time scale of thermal effects at the STM junction (∼100 ns). Hence, the use of such high-repetition-rate laser pulses bypasses the issue of thermal effects at the STM junction for low-energy (few pJ to few nJ) optical NIR pulses. It is essential to stress that all of the experiments and approaches reported here have been performed on molecules on top of metallic surfaces, where optical NIR pulses do not generate a time varying surface photovoltage unlike in semiconducting surfaces.^1,20^ The time scale of photon-induced dynamics in semiconductor surfaces will be affected by this photovoltage, but not on metallic surfaces. The temporal window in which the dynamics in single molecules can be probed by using the reported techniques spans from sub-femtoseconds to picoseconds. The step size for probing dynamics can be as small as 100 as, as the delay between pump and probe pulses is not limited by electronic jitter. A representative illustration of the temporal dynamic resolving capabilities of the two techniques can be seen in Figure 2c and Figure 4c.

The technique of frequency modulation of laser pulses involving the homodyne beating of the polarizations induced by two laser pulses with slightly different carrier frequencies has the primary limitation that it can only probe dynamics when the laser pulses are overlapping in time. The lock-in detection of the homodyne beating between the polarizations of two laser pulses is only possible when they overlap in time. However, in the frequency modulation technique, we have the possibility of imitating the polarization response of photon-induced tunneling by a single laser pulse by setting a zero delay between the two beating laser pulses. The capability of this technique enables one to probe space–time quantum fluctuations of electromagnetic fields at the STM junction.

Mechanical modulation of laser pulses leads to nonlinear heating of the nanotip of the STM junction. Nevertheless, these thermal fluctuations become negligible at high mechanical modulation frequencies (>1 kHz). It is important to note that in the pump–probe measurements described in the dispersion and polarization modulation techniques, there is a static background which comes from the difference in the tunneling current induced by short and long pulses (dispersion modulation) and between two orthogonally polarized pulse trains (polarization modulation). However, this background does not have the same origin as the background originating from thermal fluctuations of the STM junction in the amplitude modulation technique.

Ultrafast photon-induced tunneling microscopy as established by the different optical techniques described in the current work allows one to directly probe electron motion in molecules and other quantum systems. Photon-induced electron tunneling permits excitation of deterministic pathways in molecules, a characteristic feature missing in field-driven electron tunneling. Coherent control over electronic excitation will enable the driving of molecules in predetermined potential energy landscapes. We envision that it will be possible to trace electron dynamics in complex molecular systems^37^ and to map the transition state of molecules undergoing a geometrical/chemical transformation.^38^

## Methods

### STM Measurements

All experiments reported in this work were carried out with a home-built STM operating in ultrahigh-vacuum conditions (∼5 × 10^–10^ mbar) and at liquid nitrogen temperature (∼80 K). Clean Au(111) surfaces were prepared by repeated cycles of sputtering with 1.5 keV Ar^+^ ions followed by thermal annealing at 500 °C. All molecules were sublimated from a resistively heated evaporator (Dodecon). PTCDA/Au(111) samples were fabricated by sublimation of PTCDA molecules for 20 min at 400 °C. TTF-TCNQ 1:1 heteromolecular assemblies were fabricated by sublimation of TCNQ molecules for 10 min at 135 °C followed by exposure of the sample to a TTF pressure of 1 × 10^–8^ mbar for 10 s. The high vapor pressure of TTF molecules allows sublimation at room temperature.

### Laser System

The laser system used in the current work is a Ti:sapphire oscillator that produces laser pulses with a duration of <6 fs at a central wavelength of 810 nm and a bandwidth from 650 to 1050 nm. The pulses come at a repetition rate of ∼80 MHz. The laser pulses are carrier-envelope-phase (CEP) stable. Multiple reflections off two sets of chirped dielectric mirrors with different group delay dispersions (GDD = −60 fs^2^ and GDD = −120 fs^2^) were used to compensate for the positive chirp that the ultrashort pulses acquire after passing through different optical elements and air. After modulation of the laser pulses by the techniques presented in this work, the laser pulses were focused by an off-axis parabolic mirror (focal length ∼ 5 cm) to the apex of the STM tip. The diameter of the focal spot of the laser pulses is ∼30 μm.

## Supporting Information Available

(Figure S1) Characterization of the W nanotip on a Au(111) surface; (Figure S2) complete characterization measurements for the frequency modulation technique; (Figure S3) complete characterization measurements for the polarization modulation technique (PDF)
