# Supplementary material for: Ultrafast Photon-Induced Tunneling Microscopy
Source: ACS Nano. 2021 Nov 1;15(11):18071–84. doi: 10.1021/acsnano.1c06716 (PMC8613903; doi:10.1021/acsnano.1c06716)
Supplement: Supplementary file 1 — nn1c06716_si_001.pdf [file nn1c06716_si_001.pdf]

Supplementary Information

Ultrafast Photon-Induced Tunneling Microscopy

Manish Garg[1*], Alberto Martin-Jimenez[1], Yang Luo[1], Klaus Kern[1, 2]

[1] Max Planck Institute for Solid State Research, Heisenbergstr. 1, 70569 Stuttgart, Germany

[2] Institut de Physique, Ecole Polytechnique Fédérale de Lausanne, 1015 Lausanne, Switzerland

[*]Author to whom correspondence should be addressed.

[*]mgarg@fkf.mpg.de

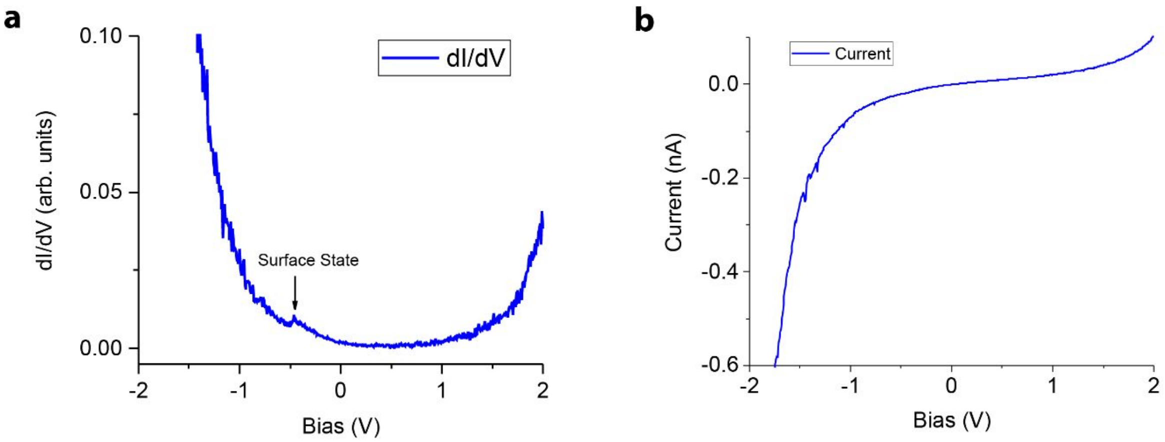

Figure S1: Characterization of the W nanotip on a Au (111) surface. (a), (b), Differential conductance (a) (dI/dV) and variation of DC tunneling current (b) as a function of the DC bias measured on a clean Au (111) surface with a W nanotip.

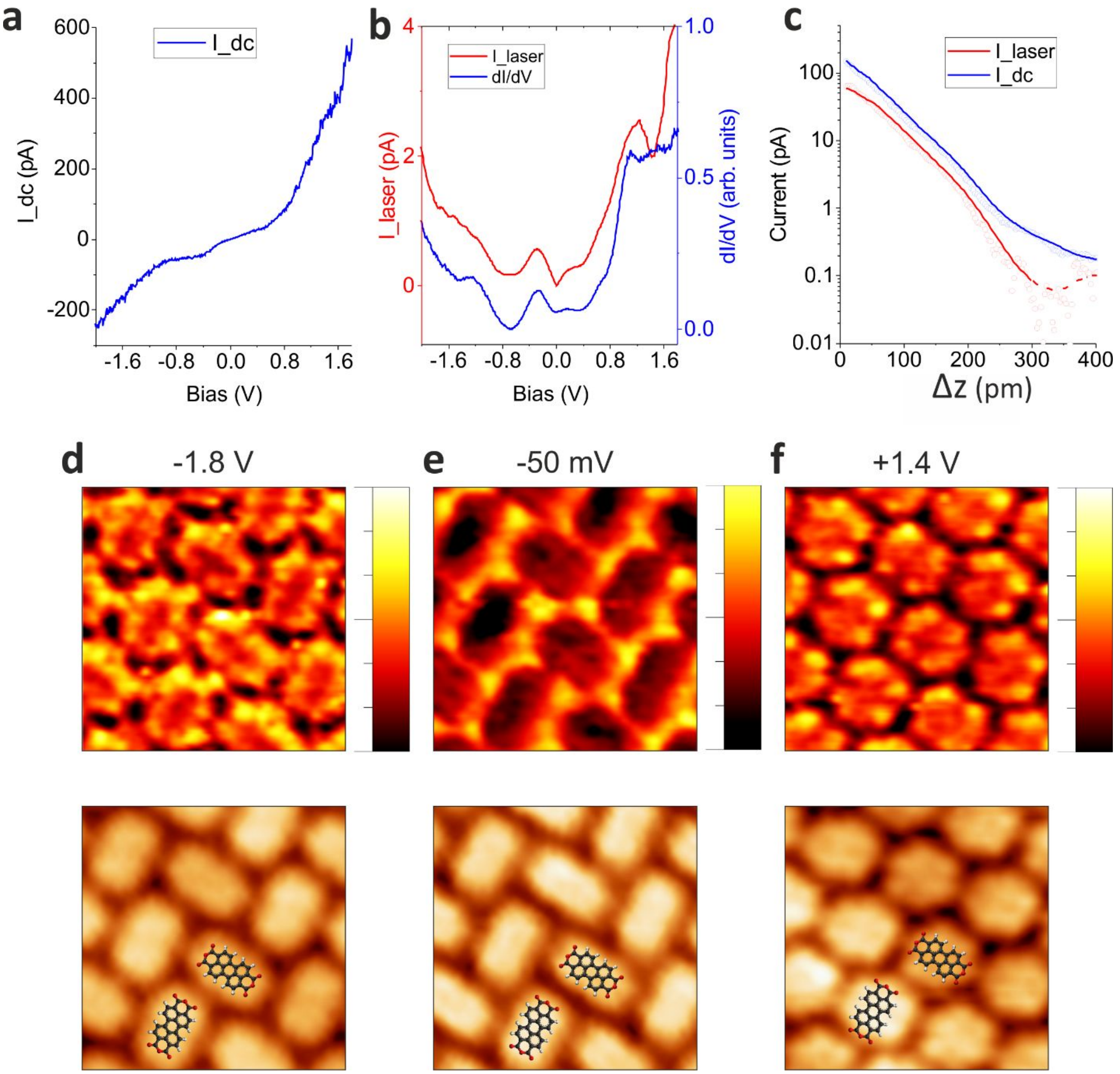

Figure S2: Frequency modulation technique. (a), (b), Measured variation of the DC tunneling current (a), dI-dV curve (blue curve) and laser-induced tunneling current (red curve) (b) as a function of the DC bias at the STM junction measured with a W nanotip on top of a PTCDA molecule constituting a monolayer assembly on Au (111). (c), Comparison of the variation of the DC tunneling current (blue-curve) and laser-induced tunneling current (red-curve) for increasing tunneling gap-widths; DC bias of 1V and 100 pA set current were used. The dashed part of the red curve represents the noise floor in the measurement. (d), (e), (f), Spatially resolved variation of the laser-induced tunneling current at various biases at the STM junction, -1.8 V, -50 mV and 1.4 V, respectively (top panels),

bottom panels: simultaneously recorded z-topography at the same DC biases. All the measurements were performed using the frequency modulation technique.

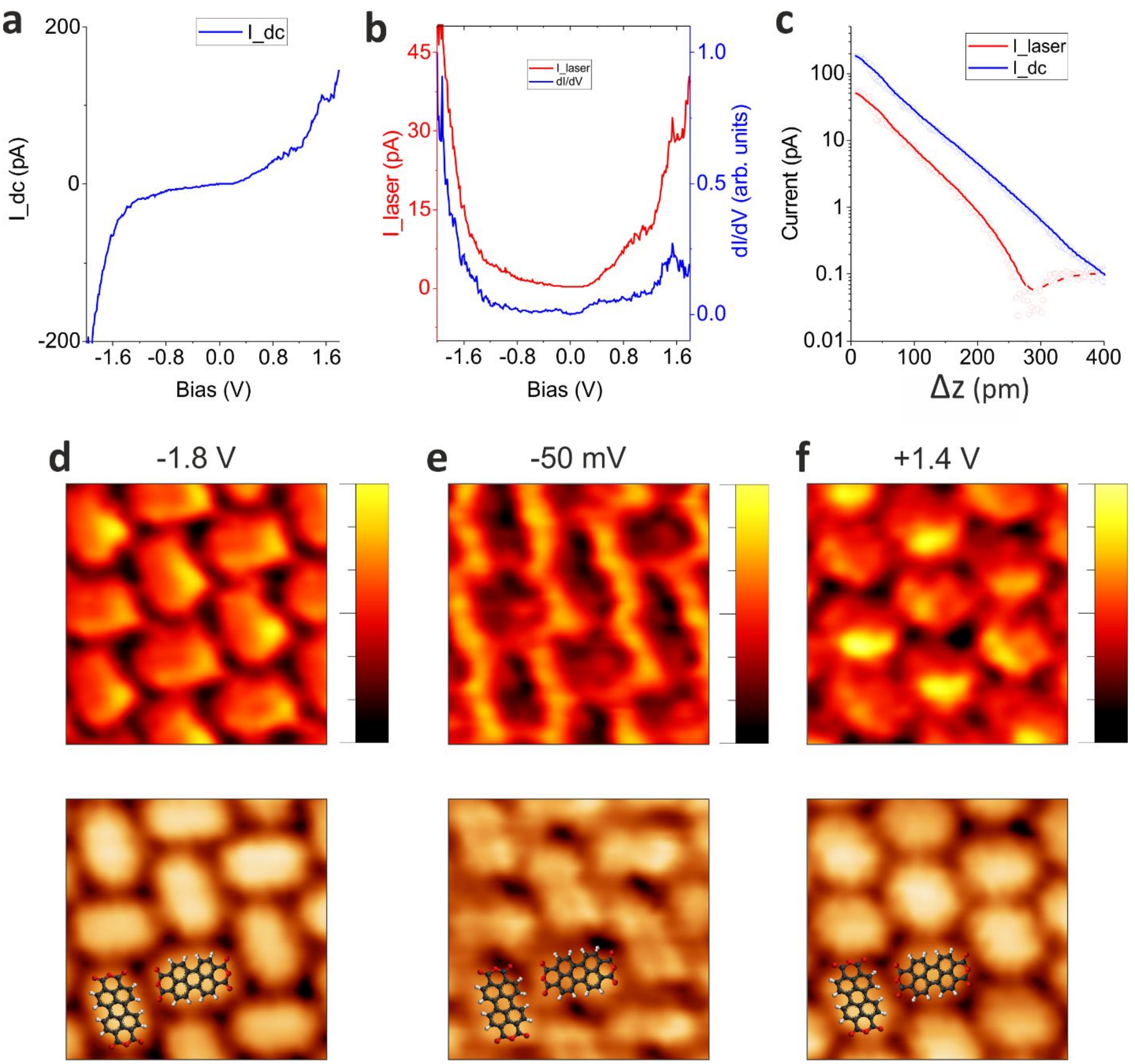

Figure S3: Polarization modulation technique. (a), (b), Measured variation of the DC tunneling current (a), dI-dV curve (blue-curve) and laser-induced tunneling current (red-curve) (b) as a function of the DC bias at the STM junction measured with a W nanotip on top of a PTCDA molecule constituting a monolayer assembly on Au (111). (c), Comparison of the variation of the DC tunneling current (blue-curve) and laser-induced tunneling current (red-curve) for increasing tunneling gap-widths; DC bias of 1V and 100 pA set current were used. The dashed part of the red curve represents the noise floor in the

measurement. (d), (e), (f), Spatially resolved variation of the laser-induced tunneling current at various biases at the STM junction, -1.8 V, -50 mV and 1.4 V, respectively (top panels), bottom panels: simultaneously recorded z-topography at the same DC biases. All the measurements were performed using the polarization modulation technique.
